# Comparing gene expression in deep infiltrating endometriosis with adenomyosis uteri: evidence for dysregulation of oncogene pathways

**DOI:** 10.1186/s12958-023-01083-9

**Published:** 2023-04-01

**Authors:** A. Marshall, K. F. Kommoss, H. Ortmann, M. Kirchner, J. Jauckus, P. Sinn, T. Strowitzki, A. Germeyer

**Affiliations:** 1 Dept. of Gynecological Endocrinology and Fertility Disorders, University Hospital Heidelberg, Im Neuenheimer Feld 440, 69120 Heidelberg, Germany; 2 Dept. of Pathology, University Hospital Heidelberg, Im Neuenheimer Feld 224, 69120 Heidelberg, Germany

**Keywords:** Gene expression analysis, Deep infiltrating endometriosis, Adenomyosis, PI3K pathway, RAS pathway

## Abstract

**Background:**

The pathogenesis of deep infiltrating endometriosis (DIE) is poorly understood. It is considered a benign disease but has histologic features of malignancy, such as local invasion or gene mutations. Moreover, it is not clear whether its invasive potential is comparable to that of adenomyosis uteri (FA), or whether it has a different biological background. Therefore, the aim of this study was to molecularly characterize the gene expression signatures of both diseases in order to gain insight into the common or different underlying pathomechanisms and to provide clues to pathomechanisms of tumor development based on these diseases.

**Methods:**

In this study, we analyzed formalin-fixed and paraffin-embedded tissue samples from two independent cohorts. One cohort involved 7 female patients with histologically confirmed FA, the other cohort 19 female patients with histologically confirmed DIE. The epithelium of both entities was microdissected in a laser-guided fashion and RNA was extracted. We analyzed the expression of 770 genes using the nCounter expression assay human PanCancer (Nanostring Technology).

**Results:**

In total, 162 genes were identified to be significantly down-regulated (n = 46) or up-regulated (n = 116) in DIE (for log2-fold changes of < 0.66 or > 1.5 and an adjusted p-value of < 0.05) compared to FA. Gene ontology and KEGG pathway analysis of increased gene expression in DIE compared to FA revealed significant overlap with genes upregulated in the PI3K pathway and focal adhesion signaling pathway as well as other solid cancer pathways. In FA, on the other hand, genes of the RAS pathway showed significant expression compared to DIE.

**Conclusion:**

DIE and FA differ significantly at the RNA expression level: in DIE the most expressed genes were those belonging to the PI3K pathway, and in FA those belonging to the RAS pathway.

## Background

Deep infiltrating endometriosis (DIE) and adenomyosis (FA) are very common benign gynecological conditions in women of childbearing age: 10–15% of women undergoing laparoscopy for benign reasons are generally found to have endometriosis [1], and in patients with infertility the incidence is even higher at 30–50% [2]. DIE is thought to occur in at least 20% of women with pelvic endometriosis [3]. FA affects 19.5% of women of childbearing age [4] [5]. In hysterectomy specimens, the incidence of diagnosed FA is 10–35% [6].

Both conditions are defined by the presence of endometrial glands and stroma either in the myometrium or outside the uterus. Originally DIE was even called “adenomyosis externa” [7].

There is a major discussion, if FA and especially DIE are related diseases or not, as in women with both entities the phenotype of FA appears to be related to the severity of endometriosis, particularly as women with DIE had a significantly higher frequency of focal adenomyosis in the external myometrium than patients with ovarian endometriosis [8]. Clinically however, the two conditions differ markedly. FA is commonly associated with dysmenorrhea, infertility, repeated implantation failure and pregnancy loss [9–11]. Multiparity and previous uterine surgery are discussed as risk factors for this condition [12–14]. DIE, on the other hand, is known to cause extensive adhesions up to complete obliteration of the Douglas space, as well as constriction of affected organs such as the bowel and bladder, and thus can cause not only dysmenorrhea but also chronic abdominal pain and, in severe cases, bowel obstruction up to the point of an ileus.

Although histologically benign, both DIE and FA are characterized by their propensity for local tissue invasion and resistance to apoptosis [15]. Notably, DIE has been described as a “benign tumor” [16]. Recent work using next-generation sequencing (NGS) has demonstrated driver mutations in cancer associated genes such as *PIK3CA*,* ARID1A*, *PPP2R1A* and *KRAS* in both, ovarian endometrioma [17] and DIE [15].

The presence of *PIK3CA*- or *KRAS*-mutated clones in histologically normal uterine endometrium in endometriosis [17] but also in patients without endometriosis has also been demonstrated [18], so the theory of the cellular origin of endometriosis requires further investigation. In contrast, the discovery of identical mutations in the *KRAS* gene in coexisting adenomyotic and endometriotic lesions in several patients [11], supports the theory of a common pathogenesis of adenomyosis uteri and endometriosis and a common molecular mechanism in these diseases [11, 19].

In this study, we aimed to further characterize the molecular mechanisms involved in FA and especially DIE to find molecular similarities and differences in both diseases. To this end, we analyzed cancer-related signaling pathways at the gene expression level using a nanostring gene panel encompassing the major signaling pathways of carcinogenesis using epithelial cells of FA and DIE. Any relevant genomic changes at the DNA level should be reflected in their gene expression and provide insight into the common or different underlying pathomechanisms of the two diseases and provide clues to the pathomechanisms of tumor development in these diseases and treatment options.

## Patients and methods

### Study population

For this study, we collected formalin-fixed and paraffin-embedded (FFPE) tissue samples for the analysis of two independent cohorts of patients with DIE or FA. Patients underwent surgery at the University Hospital, Heidelberg or cooperating clinics and the samples were histologically examined and assessed at the Dept. of Pathology of the University Hospital, Heidelberg between 2003 and 2018. Clinical records and histology were reviewed. Exclusion criteria were histological indications of cancer or dysplasia or a lesion size too small to gain sufficient material for further analysis. The samples were provided by the Tissue Bank at the National Center for Tumor Diseases (Heidelberg, Germany) in concordance with the Ethics Committee of the University of Heidelberg (approval No. S-362/2017).

### Staining and laser microdissection (LMD)

For the RNA extraction, FFPE tissue blocks from FA and DIE were selected after reviewing all original tissue slides and were recut for hematoxylin & eosin sections, to be used for reference and to determine the lesion size. RNA was extracted using 10–20 FFPE slides for each entity.

**Fig. 1:**
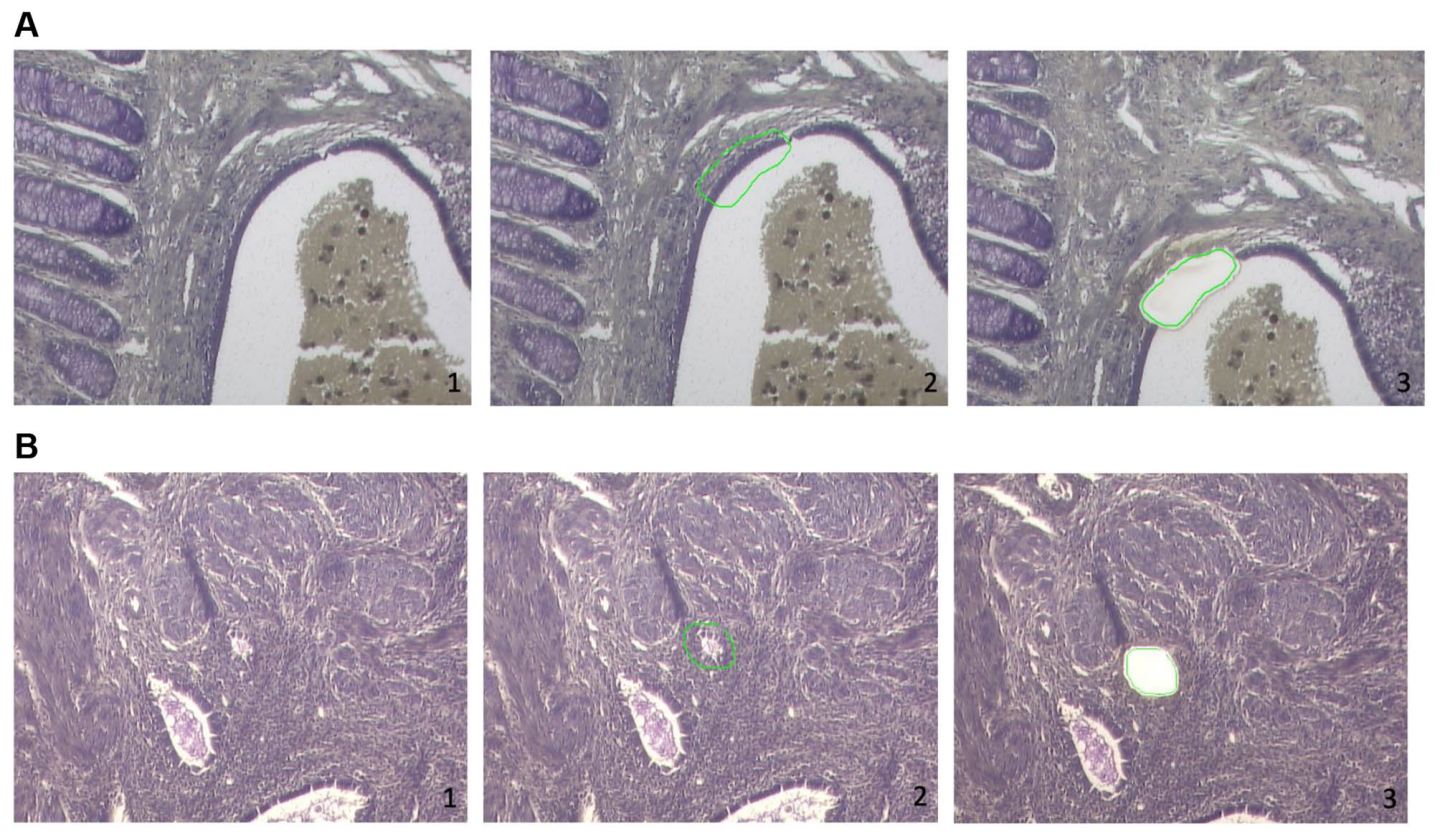
Light microscopy of deep infiltrating endometriosis of the rectum (**A**) and of adenomyosis uteri (**B**) stained with cresylviolet (10x). In each case the second image in the row shows the marking of the tissue for laser dissection [2]. The third image shows the tissue after laser dissection [3]

For mounting on Zeiss 1.0 PEN slides (Carl Zeiss, Oberkochen, Germany) and for better adhesion of the tissue to the membrane, slides were irradiated with UV light/254nm for 30 min before, FFPE tissue blocks were cut at 8 μm thickness and incubated overnight at 37 °C. They were dewaxed in xylene (100%), rehydrated through decreasing concentrations of ethanol (100, 95, 75%), stained in 1% cresyl violet acetate (Sigma-Aldrich, Taufkirchen, Germany) and again dehydrated in increasing ethanol concentrations (75, 95, 100%). After that, tissue sections were dried and stored at 4 °C. Using a ZEISS PALM LMD laser capture microdissection unit, regions of interest (epithelium of the adenomyosis or epithelium of deep infiltrating endometriosis) were microdissected. The isolated tissue fragments corresponded to an area of approximately 20.000.000 μm² for each sample. They were collected in AdhesiveCap 500 opaque tubes (Carl Zeiss) and stored at − 20 °C until further processing (Fig. 1).

### RNA isolation

Extraction of the total RNA from microdissected tissue samples was performed using the AllPrep DNA/RNA FFPE Kit (Qiagen, Venlo, the Netherlands) according to the manufacturer’s protocol. They were quantified with the Nanodrop ND-1000 spectrophotometer (NanoDrop Technologies, Rockland, DE, USA).

### Gene expression analysis

We analyzed the expression of 770 genes (Codeset: Human PanCancer Pathways) and hybridisation counts were measured using the nCounter technology (both by Nanostring™ Technology, Seattle, WA). A minimum of approximately 50 ng of total RNA was used. Hybridization time per cartridge was 16 h before measurement. According to the manufacturer’s protocol, the examined genes were attached to specific tag sequences and hybridized for 16 h at 65 °C to a capture/reporter probe pair equipped with a fluorescent barcode. These gene-specific barcodes were then detected by the nCounter Digital Analyzer providing count of genes. No cases were excluded.

The 770 genes codeset included 730 genes from 13 canonical pathways (e.g., cell cycle, chromatin modeling, apoptosis, MAPK, and PI3K) and 40 housekeeping genes [20]. The raw data were pre-analyzed for consistency using the manufacturer’s software (nSolver version 4.0). The geNorm pairwise variation statistics was used for stepwise selection of normalization genes from the housekeeping genes [21]. Six genes with minimal pairwise variation statistics were finally selected for normalization (*TLK2*, *VPS33B*, *TMUB2*, *C10orf76*, *SLC4A1AP*, *ERCC3*).

### Statistics

Differential expression analysis was carried out using a linear data model in limma [22, 23], and nominal p-values were corrected for multiple comparisons using Benjamini and Hochberg’s method [24]. All genes with an adjusted false discovery rate (FDR) of p < 0.05 and fold change of < 0.66 or > 1.5 were considered differentially expressed. Differentially expressed genes (DEGs) were subjected to functional annotation and clusterization using DAVID Bioinformatics Resources (version 6.8, https://david.ncifcrf.gov/ [25, 26]) after conversion of gene symbols to Entrez IDs and uploading to DAVID using the “RDAVIDWebService” BioConductor library [27]. Basal cytokeratin co-regulated genes were identified using DAVID analysis in the “Biological Process” category and the KEGG pathway enrichment function with a significance threshold of 0.05. The p-values of selected GO terms were corrected using Benjamini-Hochberg correction and described as adjusted p-values [24]. Otherwise, differences between samples were tested using Wilcoxon signed-rank test, and correlation was tested using Spearman’s rank correlation test, and p-value of 0.05 was considered significant. All statistical calculations were done using R version 4.0 [28]. For visualization, the R packages ComplexHeatmap and Ggplot2 were utilized.

## Results

### Study population

The study cohort included 19 female patients with DIE. DIE lesions were defined as histologically confirmed endometriosis with infiltrative growth with a depth of more than 5 mm into the wall of pelvic organs, e.g. in the bowel or bladder (Tables 1 and 2). The control group included seven patients with FA and infiltration of less than half of myometrium (superficial) or more than one half of myometrium (deep adenomyosis) (Tables 1 and 2).

**Table 1:** Cohort description including age (years), symptoms/presentation in clinic, site of manifestation, and severity according to rASRM/Enzian classification for each of the 26 patients enrolled in the study

Case	Age/ years	Symptoms/ presentation in clinic	manifestation site	rASRM/Enzian classification
1/FA	47	Tumor left ovary (12cm)	Endometriosis of the left adnexa or parametria, Adenomyosis uteri interna	ASRM III FA
2/FA	52	Bleeding disorder	Adenomyosis uteri interna	0/FA
3/FA	38	Symptomatic uterus myomatosus	Adenomyosis uteri interna	0/FA
4/FA	36	Dysmenorrhea	Adenomyosis uteri interna	0/FA
5/FA	65	Recurrent postmenopausal bleeding	Adenomyosis uteri interna	0/FA
6/FA	45	Bleeding disorder	Adenomyosis uteri interna	0/FA
7/FA	56	Bleeding disorder	Adenomyosis uteri interna	0/FA
1/DIE	33	Endoscopic findings suspicious for endometriosis in the proximal rectum	Partial sigmoid resectate with a florid, deeply infiltrating endometriosis	ASRM IV ENZIAN C3
2/DIE	46	Large endometrioma in the rectovaginal septum with vaginal pole and intestinal infiltration	(1) Endometriosis lesions in the area of the PE from the sigmoid wall (2) Extensive endometrioma in the rectal area with transition into the vaginal wall	ENZIAN A3C3
3/DIE	43	Dysmenorrhea	Partial resection of the urinary bladder with extensive tumor-like endometriosis	ENZIAN FB
4/DIE	28	Dysmenorrhea	Endometriosis lesions of the urinary bladder wall, colon wall and in the smooth muscular soft tissue	ASRM III ENZIAN A3 C3 FB
5/DIE	45	Subileus	Terminal ileum resectate with endometriosis	FI
6/DIE	44	Size-progressive mass in the area of the sigmoid colon	Partial sigmoid resection with central endometriosis	ASRM II ENZIAN C3 FO
7/DIE	30	Symptomatic endometriosis with vaginal and rectal involvement	Rectum resectate including vagina part with extensive endometriosis lesions	ASRM IV ENZIAN A3C3
8/DIE	41	Symptomatic endometriosis	Rectosigmoid with numerous, deeply infiltrating endometriosis lesions intramurally.	ASRM II ENZIAN A2C2
9/DIE	25	Deeply infiltrated endometriosis in the area of the posterior vaginal wall as well as Douglas’ space	Partial colon resection with infiltrating endometriosis.	ASRM II ENZIAN A2 C2 FO
10/ DIE	32	Deep infiltrating endometriosis of Douglas, pelvic peritoneum, bladder wall, vagina, and subphrenic on right side	(1) Vaginal wall with multiple and partially hemorrhaged, deeply infiltrating endometriosis lesions (2) Bladder wall with deeply infiltrating endometriosis	ASRM II ENZIAN A2 FB Fo
11/DIE	30	Recurrent perianal bleeding during menstruation.	Rectal resectate with multifocal manifestations of endometriosis located in the muscular wall as well as in the mesorectal adipose tissue.	ASRM II ENZIAN A1-2C2
12/DIE	36	Extensive intraperitoneal and pelvic endometriosis	1.Partial colon resectate (C. sigmoideum) with numerous endometriosis lesions 2. Ileocecal resectate with additional endometriosis lesions in the wall.	ASRM IV ENZIAN C2-3 FB FI
13/DIE	19	Dysmenorrhea	Partial colon resection with an invasive endometriosis with infiltration of the muscularis propria	ASRM II ENZIAN A2 C2 FB
14/DIE	23	Deep infiltrating Douglas endometriosis with involvement of the vagina	Douglas PE with marked, apparently deep infiltrating endometriosis.	ASRM (negative) ENZIAN A2-3 C2-3
15/DIE	34	Dysmenorrhea	Tumor-like endometriosis in the PE from the septum rectumvaginale with vaginal pole	ASRM IV ENZIAN B2-3 C2 FB
16/DIE	31	Monthly hematochezia	Resectate of the sigmoid colon with multiple endometriosis lesions of the entire intestinal wall with continuity to the intestinal lumen and serosa.	ASRM IV ENZIAN A3C3 FI
17/DIE	34	Deeply infiltrated endometriosis	Rectosigmoid resectate with extended endometriosis lesions	ASRM IV ENZIAN A3 C3
18/DIE	29	Dysmenorrhea	Recto-vaginally manifested, spreading to the rectum to the submucosa and to the vaginal wall	ASRM IV ENZIAN A3 C3
19/DIE	31 J	Known deeply infiltrated endometriosis	Infiltrating endometriosis (vaginal).	ASRM III ENZIAN A3

**Table 2:** Overview of clinical and pathological patient data

Parameter	Adenomyosis	DIE
**Patients (n)**	7	19
**Age (years)**	48.43(sd 9.42)	33.37(sd 7.58)
**BMI**	25.36(sd 5.28)	23.16(sd 3.61 unk. 3)
**Smoking history**	3 pos (4) (unk.3)	2 pos(15) (unk. 4)
**Manifestation side**		
uterus	7	0
terminal Ileum	0	1
colon	0	7
colon/rectum	0	2
rectum	0	1
rectum/vagina	0	4
vagina	0	3
bladder	0	1
**Pathology**		
**FA**	7	0
superficial	3	
deep	4	
**DIE**	0	19

The mean age of the patients suffering from FA was 48.4 years, as compared to 33.4 years for patients suffering from DIE. Patients with adenomyosis had undergone surgery due to bleeding disorders, while DIE was resected for different reasons mainly including acute and chronic pain or incipient intestinal obstruction. The GI-tract (rectum, recto-sigmoid, colon and ileum, 15 cases) was mostly affected by DIE, followed by the vagina (7 cases) and the bladder (1 case), including overlapping sites. FA included cases with superficial and deep infiltration.

### Dysregulated genes in DIE vs. adenomyosis uteri

**Fig. 2:**
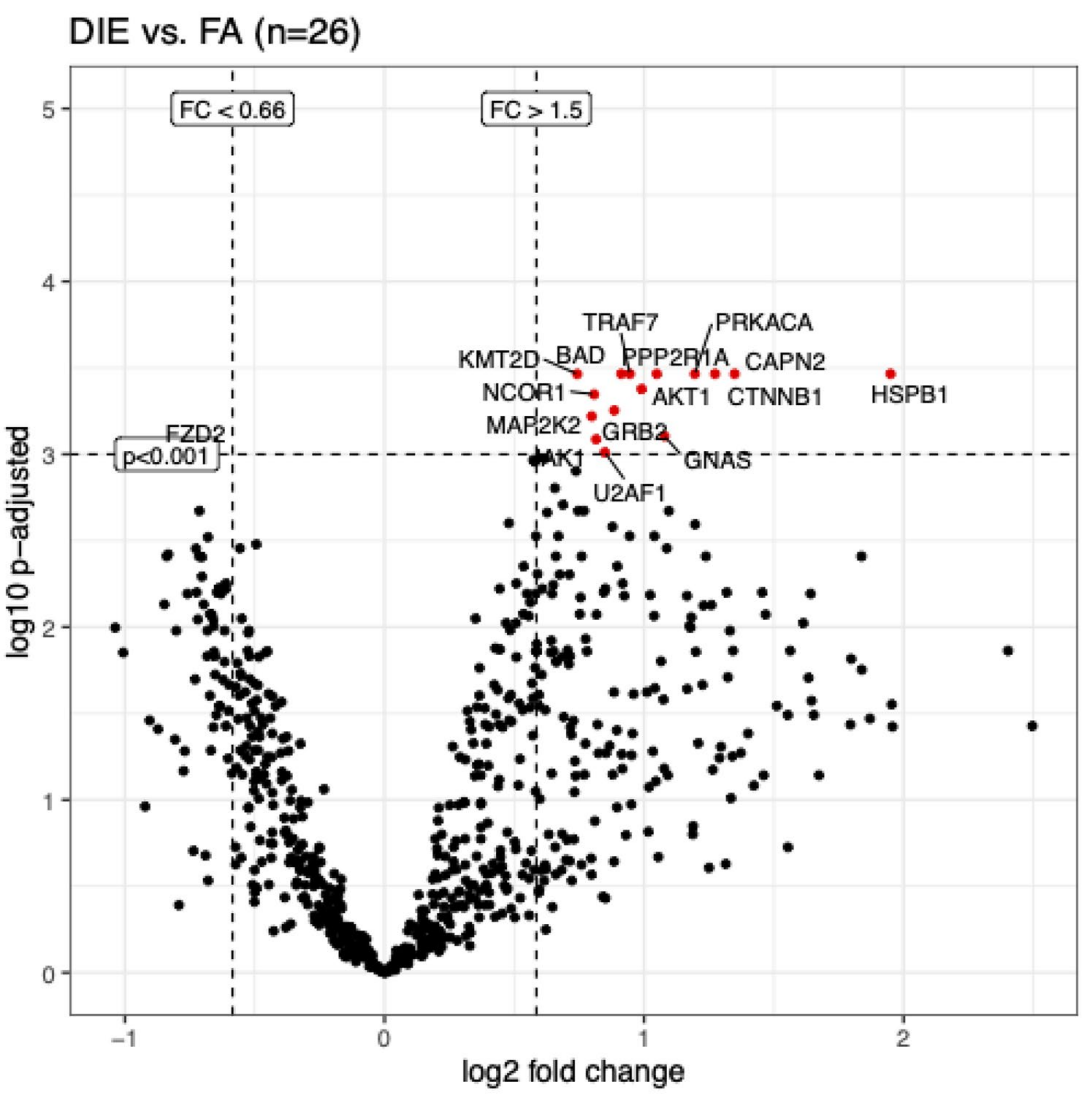
Volcano plot showing genes with most significant dysregulation in DIE vs. adenomyosis (adj. p-adjusted < 0.001 and log2 FC > 1.5). 15 genes were highly significantly upregulated in DIE, and one gene (*FZD2*) was downregulated (log2 FC < 0.66).

A mathematical model was constructed for the analysis of differential gene expression in FA and DIE. When using a threshold for fold changes (FC) < 0.66 or > 1.5, a total of 162 genes were identified that were up- or downregulated (adj. p < 0.05). This analysis included significantly more genes with upregulation in DIE (116 genes), as compared to 46 genes with relative downregulation in DIE, compared to adenomyosis (FC < 0.66). When using a stricter threshold of significance (p < 0.001 was used), 15 genes were upregulated in DIE, and only one gene (*FDZ2*) was upregulated in adenomyosis uteri (Table 3; Fig. 2). With regards to the functional properties, no specific pathway could be assigned, and therefore a separate gene ontology analysis was performed (see below).

**Table 3:** Genes with significant relative upregulation (n = 15) or downregulation (n = 1) in DIE vs. adenomyosis (> 1.5 fold, Benjamini–Hochberg adjusted P ≤ 0.001)

	Log fold change	AveExpr	t	P value	adjusted P value	B
**AKT1**	0.99	9.95	5.65	5.17E-06	4.19E-04	4.12
**BAD**	0.91	7.00	5.82	3.29E-06	3.43E-04	4.55
**CAPN2**	1.35	9.67	5.85	3.03E-06	3.43E-04	4.62
**CTNNB1**	1.27	10.53	6.11	1.54E-06	3.43E-04	5.26
**FZD2**	-0.80	6.72	-5.13	2.12E-05	9.75E-04	2.78
**GNAS**	1.08	10.65	5.28	1.40E-05	7.86E-04	3.17
**GRB2**	0.89	8.76	5.47	8.40E-06	5.57E-04	3.66
**HSPB1**	1.95	11.10	5.87	2.88E-06	3.43E-04	4.67
**JAK1**	0.82	9.24	5.24	1.57E-05	8.20E-04	3.06
**KMT2D**	0.74	8.19	5.77	3.76E-06	3.43E-04	4.42
**MAP2K2**	0.80	8.91	5.41	9.91E-06	6.03E-04	3.50
**NCOR1**	0.81	9.32	5.59	6.17E-06	4.50E-04	3.95
**PPP2R1A**	1.05	10.10	6.13	1.45E-06	3.43E-04	5.32
**PRKACA**	1.20	8.65	6.48	5.73E-07	3.43E-04	6.20
**TRAF7**	0.95	8.99	5.84	3.11E-06	3.43E-04	4.60
**U2AF1**	0.85	10.13	5.12	2.14E-05	9.75E-04	2.77

**Fig. 3:**
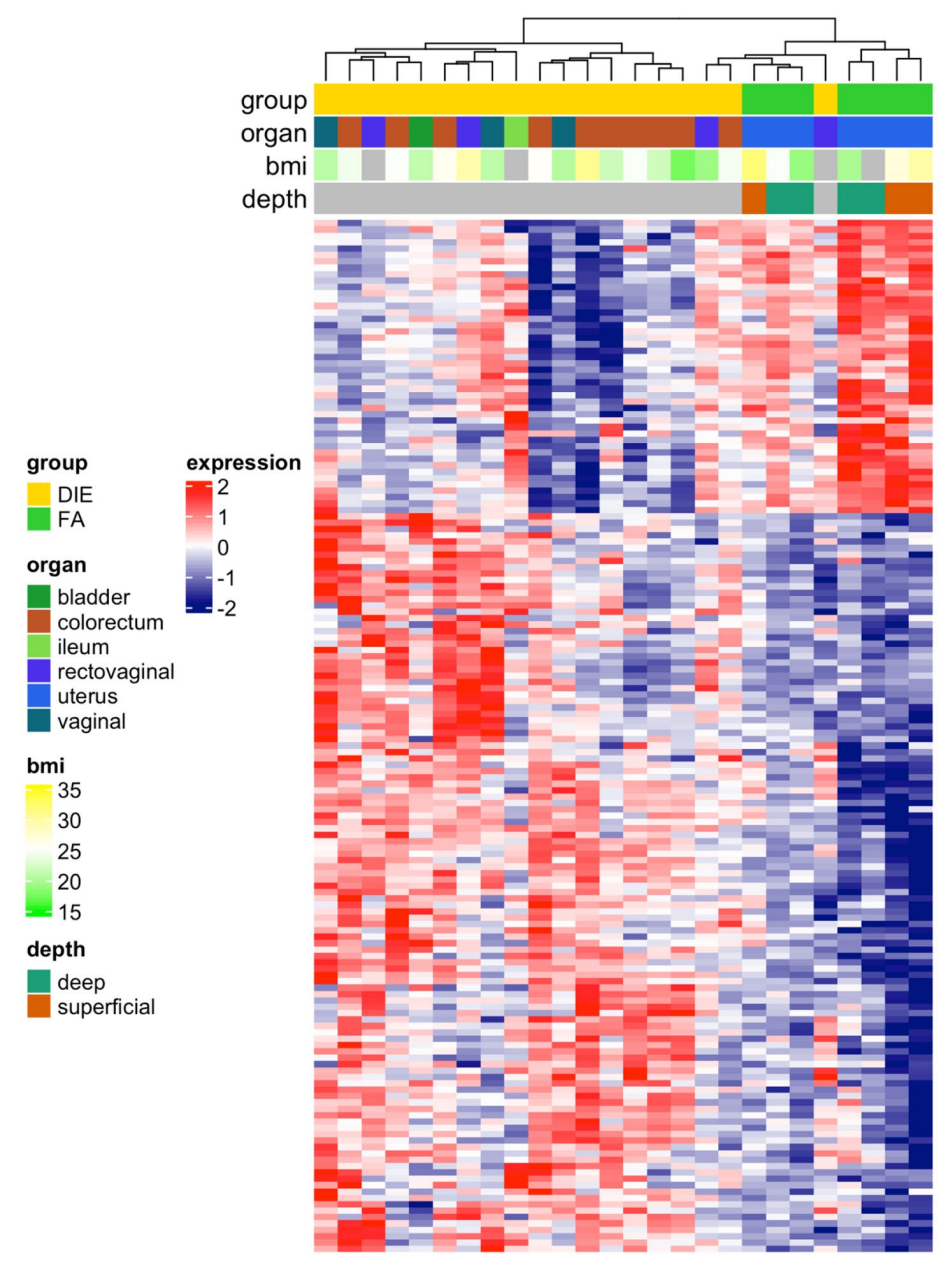
Heatmap of supervised hierarchical clustering of differential genes (adj. p < 0.05, log2 fold change > 1.5 or < 0.66 and adjusted p-value < 0.05, n = 162) for the FA versus DIE group. This includes 116 upregulated genes in DIE and 46 genes with upregulation in adenomyosis. Gene expression with cases of adenomyosis (FA) is clearly distinct from cases with deep infiltration endometriosis (DIE).

In order to relate dysregulated genes to clinical characteristics of adenomyosis and DIE, an unsupervised heatmap was constructed. Here, clustering revealed a clear separation of DIE and adenomyosis cases with only one DIE case clustering within adenomyosis (Fig. 3). But generally, samples from DIE had generally higher pathway activity scores than samples from adenomyosis (FA). In this analysis, one larger gene group with upregulation in DIE could be separated from a smaller gene group with upregulation in adenomyosis (Fig. 3). However, in this clustering no correlation of gene expression with clinical characteristics (organ, BMI, depth of adenomyosis) was evident.

### Pathways with activation in adenomyosis uteri and DIE

**Fig. 4:**
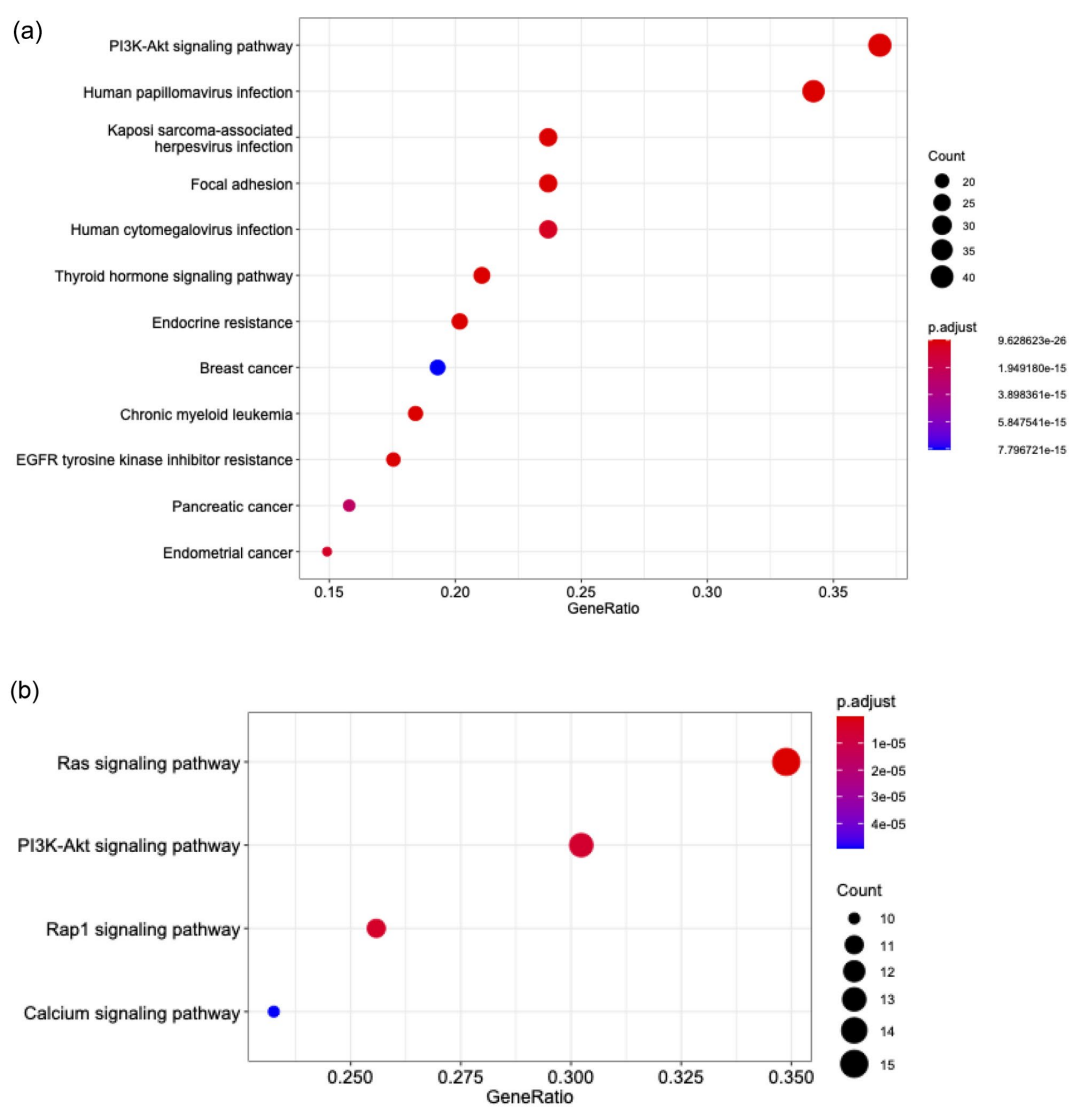
(**a**) KEGG enrichment analyses for differentially upregulated genes (p < 0.05, n = 116), 12 most significantly upregulated pathways are shown. This analysis revealed upregulating of signaling pathways in DIE, most significantly the PI3K pathway, but also pathways involved in virus infection, focal adhesion, endocrine resistance and malignancy. (**b**) Same KEGG analysis, for differentially downregulated genes (p < 0.05, n = 46), 4 most significant pathways (p < 0.0001) are shown. Here, RAS, PI3K-AKT, RAP1 and Calcium signaling pathways are significant

In order to analyze the functional properties of dysregulated genes in both diseases we performed gene ontology analysis using the KEGG pathway analysis. This analysis revealed upregulating of several signaling pathways in DIE, and interestingly, the PIK3CA pathway was most significantly upregulated. Other gene ontology groups included pathways involved in virus infection, focal adhesion, endocrine resistance and malignancy (Fig. 4a).

The identification of virus infection pathways was an unexpected finding, but further analysis revealed that these virus-pathway associated genes had 12 genes in common, and 11 of these genes were also common to the PIK3CA pathway. Therefore, it is believed that the PIK3CA pathway upregulation is the root cause for showing virus-pathway associated pathways in this analysis, and that this analysis does not point to a virus related cause of DIE. Upregulated pathways in adenomyosis included RAS, PI3K-AKT, RAP1 and calcium signaling pathways (Fig. 4b).

## Discussion

To our knowledge, this is the first study comparing isolated epithelium cells of deep infiltrating endometriosis with epithelium of adenomyosis uteri using the nanostring technology. This is of particular interest, as adenomyosis and endometriosis lesions are often surrounded by many stromal and inflammatory cells, which cause some blurring of studies at the molecular level [29]. Anglesio et al., Inoue et al. and Moore et al. have recently shown that the somatic mutation occurs in the epithelial component of DIE [15], as well as of FA [11] and in the histologically normal endometrium of healthy patients [18]. In the latter studies, analysis of the laser microdissected epithelium has been shown to yield promising results [11, 15, 17, 18, 30]. Therefore, this method was also used in the current work.

In our studies, we show for the first time that the epithelium of DIE and FA differ significantly at the RNA expression level. Interestingly, these differences in RNA expression between both entities are independent of the site of DIE manifestation and the body mass index.

Looking first at the KEGG analysis of genes whose expression is elevated in DIE compared to FA, it appears that the PI3K pathway is significantly activated. This is consistent with the results of whole-exome studies by Anglesio 2017 and Suda 2018, which detected somatic driver mutations at the DNA level in the *PIK3CA* gene in the epithelium of DIE lesions [15] and in the epithelium of ovarian endometriosis, as well as in the eutopic endometrium of healthy patients [17], among others. Since most* PIK3CA* mutations in cancers show gain of function and growth advantages [31], it was concluded that the presence of the same mutations in endometriotic epithelial cells has functional significance in the pathogenesis of the disease [17, 32]. We show that this may also be reflected in the activation of the pathway at the RNA level. Previous work has also indicated dysregulation of the PI3K pathway in endometriosis: Yin et al. 2012 demonstrated an increase in *pAKT*, albeit in stromal cells from endometriomas, compared to cells from the eutopic endometrium of healthy women, and Guo et al. 2015 also showed that phosphorylated *mTor* is increased in ectopic endometrial lesions compared to eutopic endometrium from endometriosis patients [33, 34]. Also, in a recent work by Madanes, the authors are able to demonstrate increased expression of *PI3K*, reduced expression of *PTEN*, and increased levels of *pAkt* in the ectopic and eutopic endometrium of patients with peritoneal endometriosis [35].

The PI3K-AKT-mTOR pathway is one of the most frequently dysregulated signaling pathways in carcinoma diseases [36]. Its significant activation in DIE compared to FA may explain the different behavior of the two entities under study.

We believe, that the fact, that the KEGG analysis revealed activation of virus associated gene groups (HPV, KSAHV and CMV) is due to the large intersection of genes activated in these gene groups with genes that are significantly activated in the PI3K pathway, rather than a virus-related cause of DIE, as discussed in detail above.

The focal adhesion pathway plays an essential role in cell motility, cell proliferation and cell differentiation. Its increased activation in DIE compared to FA could also explain the more progressive behavior of DIE. Interestingly, in a recent analysis of the proteome of the eutopic endometrium of endometriosis patients, Méar et al. also demonstrated an increased activation of the PI3K pathway and the focal adhesion pathway compared to healthy controls [37].

The KEGG analysis of genes that are downregulated in DIE and upregulated in adenomyosis compared to DIE shows that the RAS pathway in particular is upregulated in adenomyosis. This fits well with the findings of Inoue et al., who demonstrated a mutation in the *KRAS* gene with consecutive activation of *KRAS* in 37.1% of adenomyosis cases [11]. They detected a mutation in *PI3KCA* in only two of 70 patients, which may explain the increased expression of PI3K pathway-associated genes in DIE in our study compared with FA. In contrast, in patients with both adenomyosis and endometriosis lesions, Inoue et al. were able to detect the same *KRAS* mutation in both lesions [11]. By comparing both tissues in our study and looking at the relative gene expressions of both entities, the absolute activation of the RAS pathway is shown to be lower in endometriosis patients, which would explain why it is not detected in the KEGG analysis. In direct comparison of the two entities at the level of gene expression however, the PI3K pathway appears to be the dominant pathway in DIE and the RAS pathway in FA.

This could explain, for example, the different sensitivity of the two entities to certain therapeutic approaches. While DIE responds well to therapy with the progestin dienogest, progesterone resistance is often described in adenomyosis patients, which according to Inoue et al. is due to the *KRAS* mutations [11, 32].

If we look at the changes of single genes, it is striking that Frizzled class receptor 2 (*FDZ2*) is the only gene that is significantly upregulated in adenomyosis compared to DIE. *FZD2* is discussed as an important trigger of TGF-ß induced epithelial–mesenchymal transition (EMT) [38] and cell migration [39]. Accordingly, the induction of EMT and ultimate fibrosis by TGF-β1 appears to play a critical role in the pathogenesis of adenomyosis [40]. In addition, EMT promoted by *FZD2* also plays an important role in the metastasis of endometrial cancer [41], therefore suggesting that the invasive behavior of epithelial cells in FA has a cancerogenic aspect.

The two genes upregulated most in DIE compared to FA, Heat Shock Protein Family B (Small) Member 1 (*HSPB1*) and Calpain 2 (*CAPN2*), have not yet been described in endometriosis, despite the fact, that they play a role in proliferation and invasion in various solid tumors [42–45] and may serve this same function in DIE.

Catenin Beta 1 (*CTNNB1*), on the other hand, which is likewise upregulated in DIE epithelial cells compared to cells from adenomyosis, is discussed as a key factor in the regulation of proliferation and invasion of endometriosis [46, 47]. Since both papers demonstrate an upregulation of *CTNNB1* in endometrial stromal cells in endometriosis lesions, we can raise the question of an additional important role for *CTNNB1* action in the epithelium of endometriosis lesions.

Limitations of this study may include the following: [1] The group of patients suffering from deep infiltrating endometriosis is younger than the group of patients with adenomyosis who underwent hysterectomy. However, some of the younger patients were also on therapy with a GnRH analogue, which hormonally corresponds to a menopausal status and therefore attenuates any age-related differences. [2] By comparing adenomyosis and DIE without comparison to normal endometrium, we can only show relative expression differences, but not absolute differences compared to healthy tissue. Nevertheless, especially in view of the frequent co-occurrence of the two entities and the presumed common molecular origin, we consider our results at the gene expression level to be of further value, particularly with regard to possible different therapeutic approaches. [3] The sample size is relatively small with 7 patients with adenomyosis and 19 patients with DIE. Further studies with a larger number of patients to evaluate the possible influence of age, therapy concept or site of manifestation of DIE would be useful.

## Conclusions

Deep infiltrating endometriosis and adenomyosis uteri differ significantly at the RNA expression level: for deep infiltrating endometriosis, the genes most expressed were those belonging to the PI3K pathway, and for adenomyosis, those belonging to the RAS pathway.

## Data Availability

Data or material are available on reasonable request.

## Abbreviations

ARID1A: AT-rich interaction domain1A
BMI: Body mass index
C10orf76: chromosome 10, open-reading frame 76
CAPN2: Calpain 2
CMV: cytomegalovirus
CTNNB1: Catenin Beta 1
DEG: differentially expressed genes
DIE: deep infiltrating endometriosis
DNA: deoxyribonucleic acid
EMT: epithelial mesenchymal transition
ERCC3: ERCC excision repair 3
FA: Adenomyosis uteri
FC: Fold change
FDR: False discorvery rate
FFPE: formalin-fixed and paraffin-embedded
FZD2: Frizzled class receptor 2
GI: Gastrointestinal tract
GO: Gene ontology
HPV: Human papillomavirus
HSPB1: Heat Shock Protein Family B (Small) Member 1
KRAS: KRAS Proto-oncogene, GTPase
KSAHV: kaposi sarcoma associated herpesvirus infection
LMD: laser microdissection
NGS: next-generation sequencing
pAKT: phosphorylated AKT Serine/Threonine Kinase
PI3K: Phosphatidylinositol 3-kinase
PI3KCA: Phosphatidylinositol-4,5-Bisphosphate 3-Kinase Catalytic Subunit Alpha
PPP2R1A: Protein phosphatase 2, regulatory subunit A
PTEN: Phosphatase and Tensin Homolog
RAP1: Ras-related protein 1
RAS: Rat sarcoma
rASRM: Revised American Society for Reproductive Medicine classification
RNA: Ribonucleic acid
SLC4A1AP: Solute Carrier Family 4 Member 1 Adaptor Protein
TGF-ß: Transforming growth factor ß
TLK2: Tousled Like Kinase 2
TMUB2: Transmembrane and Ubiquitin Like Domain Containing 2
VPS33B: Vacuolar Protein Sorting-Associated Protein 33B
